# Synergistic Inhibition of Endothelial Cell Proliferation, Tube Formation, and Sprouting by Cyclosporin A and Itraconazole

**DOI:** 10.1371/journal.pone.0024793

**Published:** 2011-09-28

**Authors:** Benjamin A. Nacev, Jun O. Liu

**Affiliations:** 1 Department of Pharmacology and Molecular Sciences, Johns Hopkins University School of Medicine, Baltimore, Maryland, United States of America; 2 Medical Scientist Training Program, Johns Hopkins University School of Medicine, Baltimore, Maryland, United States of America; 3 Department of Oncology, Johns Hopkins University School of Medicine, Baltimore, Maryland, United States of America; University of Frankfurt - University Hospital Frankfurt, Germany

## Abstract

Pathological angiogenesis contributes to a number of diseases including cancer and macular degeneration. Although angiogenesis inhibitors are available in the clinic, their efficacy against most cancers is modest due in part to the existence of alternative and compensatory signaling pathways. Given that angiogenesis is dependent on multiple growth factors and a broad signaling network *in vivo*, we sought to explore the potential of multidrug cocktails for angiogenesis inhibition. We have screened 741 clinical drug combinations for the synergistic inhibition of endothelial cell proliferation. We focused specifically on existing clinical drugs since the re-purposing of clinical drugs allows for a more rapid and cost effective transition to clinical studies when compared to new drug entities. Our screen identified cyclosporin A (CsA), an immunosuppressant, and itraconazole, an antifungal drug, as a synergistic pair of inhibitors of endothelial cell proliferation. In combination, the IC_50_ dose of each drug is reduced by 3 to 9 fold. We also tested the ability of the combination to inhibit endothelial cell tube formation and sprouting, which are dependent on two essential processes in angiogenesis, endothelial cell migration and differentiation. We found that CsA and itraconazole synergistically inhibit tube network size and sprout formation. Lastly, we tested the combination on human foreskin fibroblast viability as well as Jurkat T cell and HeLa cell proliferation, and found that endothelial cells are selectively targeted. Thus, it is possible to combine existing clinical drugs to synergistically inhibit *in vitro* models of angiogenesis. This strategy may be useful in pursuing the next generation of antiangiogenesis therapy.

## Introduction

Angiogenesis, the process of new blood vessel growth and development, underlies a number of human diseases including cancer, macular degeneration, psoriasis, rheumatoid arthritis, diabetic retinopathy, and pulmonary hypertension [1]. Inhibitors of angiogenesis such as the anti-VEGF antibody bevacizumab are used clinically to treat cancer. However, the experience with existing therapies has been mixed. While they have shown efficacy, their effects in terms of halting disease progression and improving survival have been modest and often involve side effects including hypertension and increased risk of stroke [2]. Hence, although the promise of angiogenesis inhibitors has been demonstrated, there is a clear need for more effective anti-angiogenic therapies.

On average, the development of a clinically viable drug requires an investment of roughly $800 million and takes about 12 years [3]. One strategy to accelerate drug development is to re-purpose existing drugs [4]. Because re-purposed drugs have already been approved for clinical use, their pharmacodynamic and pharmacokinetic properties are well established. In addition, existing drugs have acceptable levels of toxicity and in many cases they have known mechanisms, which makes their pharmacology amenable to detailed molecular study. Thus, by focusing on existing drugs, many hurdles in drug development are already cleared. The end result is a drastically shortened path from bench to bedside when old drugs are discovered to have new applications. We have previously adopted this approach when we assembled and screened the Johns Hopkins Drug Library (JHDL) for inhibitors of angiogenesis and other activities [5]–[9]. Presently, the JHDL contains ∼3,300 drugs approved by the US Food and Drug Administration or foreign equivalent. The initial screen for angiogenesis inhibitors identified 221 compounds with >50% inhibition of human umbilical vein endothelial cell (HUVEC) proliferation at a 10 µM dose.

A number of these hits had IC_90_ doses above the peak plasma level obtained under clinical dosing regimens or had dose-limiting toxicities. One way to expand the clinical applicability of these hits, we reasoned, was to find synergy between them, thereby reducing the doses needed for those synergistic pairs to inhibit angiogenesis *in vivo*. We have thus conducted a screen for synergy among 741 binary combinations of 39 clinical drugs that were hits from the initial screen. In addition to lowering the necessary dose of otherwise toxic agents, combination therapy is often used to limit the potential for drug resistance and to achieve synergistic inhibition of multiple independent pathways that converge on a single essential molecular process. For these reasons, the simultaneous use of multiple drugs has been an effective strategy to overcome diseases intractable to single agent therapies. For instance, combination therapy is now the standard of care in treating HIV infection and many neoplasms [10].

Synergy, or superadditivity, is often observed upon the inhibition of multiple pathways that converge to promote a single biological process such as proliferation. Thus, it is hypothetically possible that synergistic inhibition of angiogenesis may be possible given that signaling in angiogenesis is complex and involves multiple pathways including those downstream of growth factors such as vascular endothelial growth factor (VEGF) and basic fibroblast growth factor (bFGF) [11], [12]. In fact, the limitations of anti-VEGF therapy have been attributed to the existence of redundant alternative pathways in the *in vivo* pro-angiogenic signaling network [13]. Thus, a more effective strategy to inhibit angiogenesis may be to simultaneously target multiple pathways. Just as anticancer regimens have evolved to simultaneously utilize drugs with multiple mechanisms to achieve synergy, so might anti-angiogenic regimens have to evolve to provide additional efficacy. Thus, we sought to determine whether there exist clinical drugs that synergistically inhibit endothelial cell proliferation and tube formation.

## Materials and Methods

### Reagents and materials

Pooled HUVEC and EGM-2 bullet kit media were purchased from Lonza. Jurkat T cells (a human acute T cell leukemia line) and HeLa cells (a human cervical adenocarcinoma line) were from the American Type Tissue Collection. Low and high glucose DMEM, RPMI 1640, fetal bovine serum, and penicillin/streptomycin were from Gibco. Recombinant human VEGF_165_ and bFGF_146_ were purchased from R&D systems and reconstituted in 0.1% BSA in PBS as 100 µg/mL and 10 µg/mL stocks, respectively. Methyl cellulose (4 cP) was purchased from Sigma and used to prepare methocel as previously described [14]. Itraconazole (Ita) (Sigma), cyclosporin A (CsA) (LC labs), and sunitinib (LC labs) were stored frozen in DMSO and added to cells from 200× stocks. Calcein AM and Alamar Blue were purchased from Invitrogen and [^3^H]-thymidine was from PerkinElmer. Glass filtermats were obtained from Wallac. Phenol red free Matrigel and rat tail collagen type I were from BD biosciences.

### Cell culture

All cells were grown at 37°C with 5% CO_2_ in a humidified environment. HUVEC were grown in EGM-2 bullet kit media and used between passages 2 and 8. Jurkat T cells were grown in RMPI 1640 (+10% FBS, 1% penicillin/streptomycin), HeLa were grown in low glucose DMEM (+10% FBS, 1% penicillin/streptomycin), and HFF in high glucose DMEM (+10% FBS, 1% penicillin/streptomycin).

### Proliferation assays

2000 HUVEC or HeLa/well or 1×10^4^ Jurkat T cells/well were seeded in a 96-well plate (Costar) in 199 µL media. After an overnight recovery, drugs were added. For CsA+Ita combinations the molar ratio was always 10∶1. Following a 24-h incubation, cells were pulsed with 0.9 µCi of [^3^H]-thymidine for 6 h, washed once with PBS, trypsinized, and transferred to filtermats (Wallac) using a Mach III M Harvester 96 (Tomtec). For Jurkat T cells, the PBS wash and trypsinization steps were omitted. After drying, [^3^H]-thymidine retention on the filtermats was determined by scintillation counting using a 1450 Microbeta apparatus (Wallac). Counts were normalized to that of control cells treated with vehicle only. GraphPad Prism (v4.03) software was used to determine IC_50_ values using a four parameter logistic regression.

In the case of growth factor-dependent proliferation assays, the cells were first seeded as above but in basal EBM-2 basal media (Lonza) with 2% FBS added (hereafter referred to as ‘basal’ media). After an overnight recovery, the media was replaced with either basal media, standard EGM-2 media, basal media with 100 ng/mL VEGF_165_ or basal media with VEGF_165_ vehicle alone. Drugs were then added and the assay was continued as described above. In processing the data for VEGF-dependent proliferation, the [^3^H] counts for basal proliferation at each dose was subtracted from that of basal+VEGF proliferation prior to normalizing the data to vehicle control.

### Cell Viability Assay

HUVEC and HeLa were plated at 2000 cells/well and HFF at 2500 cells/well in a 96-well plate in 199 µL of media. Cells were incubated with drug for 30 h and then washed with 200 µL of PBS and incubated for 30 min at 37°C with 100 µL of 1 µM Calcein AM diluted from a 1 M stock in DMSO. The excess dye solution was then aspirated and 100 µL PBS was added prior to reading with a fluorescence plate reader (Fluostar Optima). The fluorescence of drug treated samples was normalized to DMSO controls. Three independent experiments were performed.

An Alamar Blue assay for Jurkat viability was used since it does not require the media to be removed prior to analysis, which is advantageous for cells grown in suspension. 1×10^4^ Jurkat cells were seeded per well of a 96-well plate in 200 µL of media. Following a 24-h incubation with drug, 20 µL of Alamar blue was added and cells were incubated for an additional 6 h. The florescence of each well was measured at 590 nm after a 544 nm excitation. Media only wells were included as blanks. Three independent experiments with multiple technical replicates were performed.

### Tube formation assay

For the synergy analysis, 230 µL of ice cold, phenol red-free matrigel was added to a 24-well plate using a chilled pipet tip. Following a 30–40 min incubation at 37°C, 7×10^4^ HUVEC were added in 500 µL of media. Cells were treated with drug for 18 h. The cells were then gently washed with 500 µL PBS and followed by incubation with 300 µL of 2 µM calcein AM (diluted in PBS from a 1 M DMSO stock) for 30 min. The calcein AM was replaced with 500 µL PBS and the tube network was photographed using fluorescent microscopy. For the comparison of sunitinib and CsA+Ita, the above experiment was scaled down to a 96-well plate format. 50 µL of ice cold matrigel was used and 1.5×10^4^ HUVEC in 200 µL of media were seeded. Following an 18-h drug treatment, the cells were washed with 150 µL of PBS and incubated for 30 minutes at 37°C with 100 µL of Calcein AM which was replaced with 100 µL of PBS prior to imaging. In both cases, the images were inverted and equal areas of the central or best focused region of the image were cropped out using Adobe Photoshop (v 9.0.2). The tube networks were quantified using Angioquant [15].

### Sprouting Assay

This assay was based on work by Korff and Augustin [16]. We modified the procedure by first coating the bottom of the tissue culture surface with a collagen, growth factor, and drug solution to ensure that the spheroids did not contact the tissue culture plate. First, VEGF_165_ (30 ng/mL) and bFGF (30 ng/mL) or vehicle (0.1% BSA in PBS) was added to a methocel∶media (42∶58) solution, followed by drug or vehicle addition and mixing. This was then mixed 1∶1 (0.5 mL total volume) with a working collagen solution [3.5 mg/mL collagen (10 equivalents) and ice cold 10× PBS (1 equivalent) premixed with 1 N NaOH (0.23 equivalents), kept on ice]. Immediately after mixing, the matrix was added to a pre-warmed 24-well plate (37°C) and returned to 37°C. Meanwhile, HUVEC which were induced to form spheroids of 1000 cells each by suspension in 25 µL media∶methocel (4∶1) hanging drops in uncoated sterile petri dishes for 18 h were washed from the plates in PBS and pelleted at 150×*g*, no brake. The spheroids were suspended in serum free EGM-2 (∼55 spheroids/mL) and transferred to Eppendorf tubes in 1-mL aliquots and pelleted. The supernatant was carefully aspirated and the tubes were gently scraped to release the pellet. The media∶methocel solution with growth factors was layered over the pellet (0.25 mL) followed by the addition of 0.25 mL working collagen stock and then drugs or vehicle. After mixing, the solution was immediately transferred to the plates containing the base coat of matrix. Assuming a 100% yield, approximately 55 spheroids were seeded per well. After a 30-minute incubation, 150 µL of EGM-2 with 2% FBS was added to the surface of each well. After a 24-h period, the sprouts were imaged using an Olympus BX61 microscope with a 20× phase I contrast objective. Eight spheroids in 4 independent experiments were measured for each condition. Cumulative sprout length (per spheroid) was quantitated using Volocity software (v 5.4.1; PerkinElmer). Quantitation was optimized with a package of Volocity modules for intensity filtering (to highlight the sprouts), exclusion of objects <100 pixels^2^, hole filling, noise reduction (very coarse filter), skeleton measurement and exclusion of sprouts fragments smaller than 60.5 µm.

### Synergy calculations and Statistical analysis

The combination indices and dose reduction index (DRI) values were calculated using CompuSyn. In the case of the proliferation assays, the synergy parameters were skewed towards zero. Therefore, the data was log transformed to normalize the distribution prior to performing Student's t-test. Otherwise, Student's t-test was applied to untransformed data. In comparing the potency of CsA + Ita with the same dose of sunitinib in tube formation assays conducted in parallel, paired t-tests were used. In all hypothesis testing for synergy, H_0_ = additive or antagonistic interactions. For DRI, H_0_ = unchanged or increased dosing requirements. For other applications, the t-test was 2-sided.

## Results

### Design of the Synergy Screen

For the synergy screen, we selected a pool of thirty-nine drugs from among the initial list of 221 hits generated by a prior screen of the JHDL for HUVEC proliferation inhibition (Table 1) [5], [6]. This pool was chosen to exclude topical or generally cytotoxic drugs, and to represent a large number of different drug classes in order to increase diversity. This was done to maximize the coverage of targeted molecular pathways so as to increase the probability of identifying unpredicted connections between drug targets. In all, twenty different drug classes were chosen to include classes as diverse as antiviral, vasodilator, and antihistamine drugs (Table 2).

**Table 1:** Collection of clinical drugs screened in binary combinations for synergistic inhibition of HUVEC proliferation.

Albendazole	Clomiphene	Fluvastatin	Miconaozole	Simvastatin
Amphotericin B	Cyclosporin A	OH-progesterone	Mycophenolic acid	Terconazole
Atorvastatin	Digoxin	Hypericin	Nimodipine	Terfenadine
Atovaquone	Emitine	Idoxuridine	Pentamidine	Thiabendazole
Auranofin	Ergosterol	Iodoquinol	Pimecrolimus	Tolonium
β-estradiol	Ethinyl esterdiol	Itraconazole	Progesterone	Tribromosalan
Bromocriptine	Felodipine	Lovastatin	Raloxifene	Zidovudine
Chlortetracyclin	Floxuridine	Mebendazole	Sertraline	

**Table 2:** Distribution of Drug Classes.

Drug Class	Frequency
Anthelminthinc	3
Antiamebic	1
Antibacterial	3
Antibiotic	1
Antidepressant	2
Antifungal	3
Antihistamine	1
Antihyperlipidemic	5
Antihypertensive	1
Antirhematic	1
Antiviral	4
Bone Metabolism Modulator	1
Cardiotonic	1
Estrogen	3
Hemostatic	1
Immunosuppressant	3
Pituitary Modulator	1
Proestrogen	2
Vasodilator	1
Vitamin	1

The pool was combined in 741 unique two-drug combinations and screened for the ability to synergistically inhibit the proliferation of HUVEC. Since *in vivo* angiogenesis requires endothelial cell proliferation and cell proliferation is amenable to high-throughput analysis, proliferation is often used as a proxy for angiogenesis. The screening was conducted in multiple phases (Figure S1). In the first phase, the potency of each drug concentration was determined in duplicate for combinations of the drugs at 1-, 0.5-, and 0.25-fold of the rough IC_50_ doses of each drug alone. Proliferation was measured by the incorporation of [^3^H]-thymidine during a 6-h pulse following a 24-h drug treatment. The presence of synergy was determined using the Chou-Talalay analysis, which required the side-by-side generation of the individual drug dose response curves along with the combination proliferation data [17]. The Chou-Talalay method uses the median effect equation to determine if the combination of two drugs produces an effect which exceeds that predicted by the simple addition of the individual drug effects [17], [18]. The key parameter returned by this analysis is the combination index (CI). A CI of 1 indicates pure additivity; a CI greater than 1 indicates antagonism; and a CI between 0 and 1 indicates synergy. The initial screening phase identified 47 pairs with a CI in the synergistic range.

In Phase 2 of the screen, we attempted to validate the initial 47 hits by performing complete dose-response curves consisting of seven doses for each combination. This allowed for a more compete characterization of the synergy since a broader dose range produces data that is resistant to random fluctuations in the high throughput format. The Chou-Talalay analysis was again applied and drug combinations that produced statistically significant synergy (i.e. p<0.05) across a range of effect levels (i.e. IC_30_–IC_60_) were considered validated hits. Forty-six of the initial 47 hits did not meet this validity test and were discarded as false positives.

### Cyclosporin A and Itraconazole Synergistically Inhibit Endothelial Cell Proliferation

**Figure 1:**
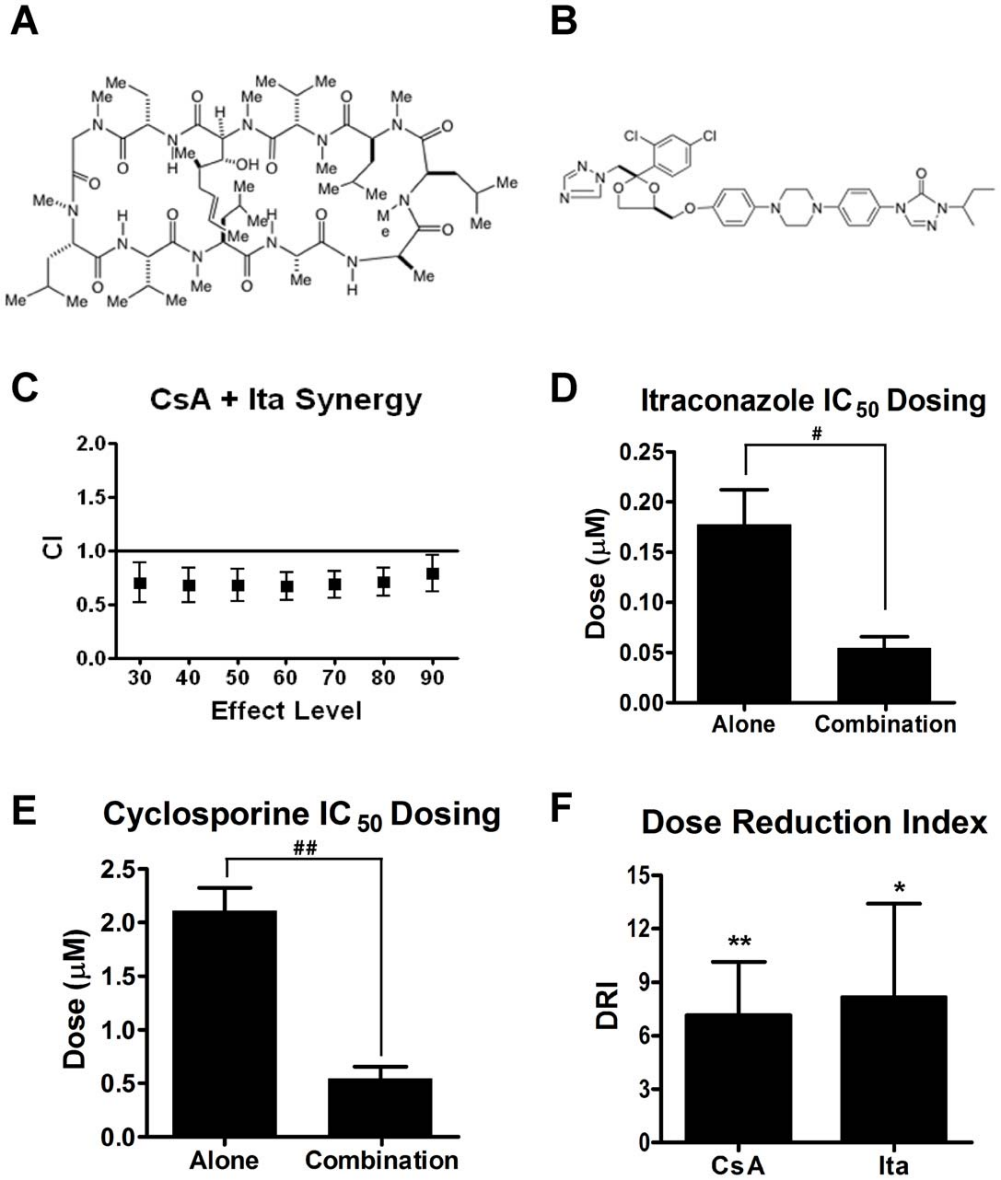
Cyclosporin A and itraconazole are synergistic inhibitors of HUVEC proliferation. Chemical structures of cyclosporin A (A) and itraconazole (B). The CI plot for CsA+Ita indicated CI in the synergistic range across a wide range of effect levels (C). The IC_50_ dose of both Ita (D) and CsA (E) is significantly reduced in combination. This is also reflected in the dose reduction index (F). Bars, standard error of the mean (SEM). n = 8; * p<0.1; ** p<0.05; # p<0.005; ## p<0.0001.

The second phase of screening identified two commonly used clinical drugs, cyclosporin A (CsA), an immunosuppressant, and itraconazole (Ita), an antifungal drug, as synergistic inhibitors of HUVEC proliferation (Fig. 1, A and B). The CI value is dependent on the effect level. Therefore we calculated the CI value across a range of effects and performed a test for statistical significance for each data point (Fig. 1C; Table 3). The CsA + Ita combination was synergistic over a range from IC_30_ to IC_90_ with the strongest statistical significance correlating with points in the IC_30_ to IC_60_ range.

**Table 3:** Combination Indices and p-values versus effect level for CsA and Ita combination treatment.

IC Level	CI Value	p-value
30	0.7	0.043
40	0.68	0.041
50	0.67	0.045
60	0.67	0.048
70	0.68	0.059
80	0.71	0.073
90	0.79	0.104

Effective drug combinations result in a marked decrease in the concentration of each drug necessary to produce the desired effect when compared the doses of each drug to produce the same effect as a single agent. This dose reduction is amplified in the case of a synergistic interaction. Based on the HUVEC proliferation IC_50_ doses for CsA, Ita, and the combination regimen, the combination of the two drugs resulted in a statistically significant 4-fold and 3-fold reduction in the CsA and Ita dose, respectively (Fig. 1, D and E). This resulted in combination IC_50_ doses of 540 nM (CsA) and 54 nM (Ita). We validated these findings using a modification of the CI equation to yield the Dose Reduction Index (DRI), which also indicates the fold reduction of a drug dose in combination [17]. In this analysis, the DRI for CsA was 7.1-fold (p<0.05) and for Ita was 8.2-fold (p<0.1) (Fig. 1F).

**Figure 2:**
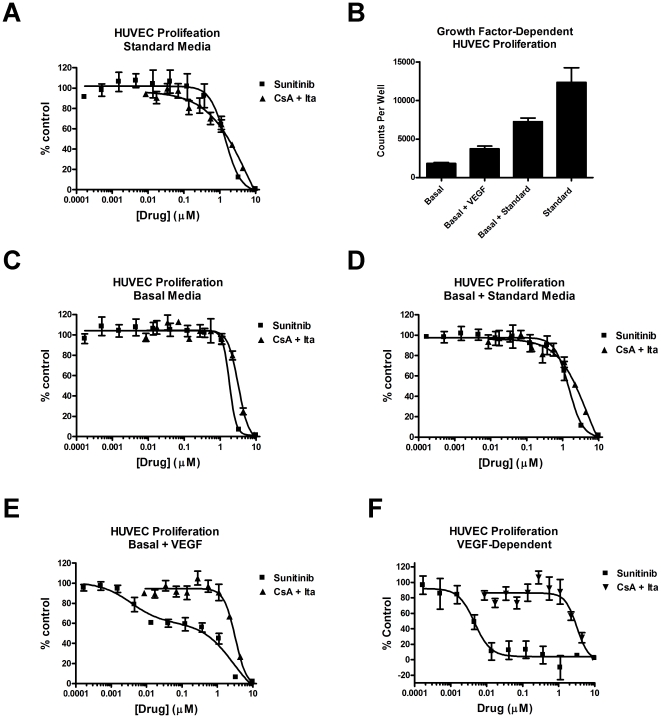
The potency of cyclosporin A and itraconazole against HUVEC proliferation is independent of VEGF signaling. (A) The proliferation of HUVEC grown in standard media in the presence of the indicated doses of CsA + Ita or sunitinib was determined. (B) HUVEC were seeded in either standard media (standard), or basal media (EBM-2+2% FBS), which was changed the next day to either fresh basal media with 100 ng/mL VEGF (basal + VEGF) or VEGF vehicle (basal), or standard media (basal + standard). The cells were then treated with drug vehicle for 24 h and the incorporation of [^3^H]-thymidine after a 6 h pulse was determined. The dose response curves CsA + Ita and sunitinib were determined in basal media (C), basal + standard media (D), and basal media + VEGF (E). (F) The dose-response curve for VEGF-dependent proliferation (basal media + VEGF proliferation minus basal media proliferation) was also determined. Bars = SEM, n = 3.

Having shown that the CsA+Ita combination was synergistic for inhibition of HUVEC proliferation, we compared the combination to sunitinib, an FDA-approved antiangiogenic drug (Fig. 2) [19]. Both the combination and sunitinib inhibited HUVEC proliferation with an IC_50_ in the single-digit micromolar range (Fig. 2A), which was unexpected because sunitinib is known to inhibit VEGFR2, the major receptor for VEGF-mediated angiogenic signaling, with a K_i_ of 9 nM [20]. However, a recently study concluded that a number of growth factors, some of which are present in standard HUVEC media, including FGF2 and EGF, can rescue the effects of sunitinib on HUVEC proliferation [21]. Thus, in standard media the discrepancy between the HUVEC proliferation IC_50_ and the K_i_ for the VEGFR2 receptor was likely due to the influence of growth factors other than VEGF. Presumably, the less potent activity of sunitinib against these growth factor receptors and other off-target effects dictated the observed IC_50._

To address this possibility, we made a further comparison between the potency of CsA+Ita and sunitinib for inhibiting HUVEC first grown in basal media (EBM-2+2% FBS) and then stimulated with 100 ng/mL VEGF_165_ (Fig. 2, B, C and E). As a control, cells grown in basal media alone or grown first in basal media and then standard media were also tested (Fig. 2, B and D). In the absence of exogenous growth factors, the IC_50′_s for CsA + Ita and sunitinib were remarkably similar and in the single-digit micromolar range as in the experiments with standard media. This is consistent with the findings of several other groups in similar experiments [22], [23]. However, when HUVEC were treated in media supplemented with VEGF, the potency of CsA + Ita was lower than that of sunitinib (Fig. 2E). Interestingly, the dose-response curve for sunitinib had two apparent EC_50_s, one corresponding to the IC_50_ seen in either standard or basal media and a second much lower EC_50_. This mid-curve plateau has also been observed by other groups [23]. We reasoned that the observed curve was the superimposition of sunitinib's effects on both VEGF-dependent and -independent HUVEC proliferation. Thus, we subtracted the proliferation in basal media at each dose of both sunitinib and CsA + Ita from that in VEGF-supplemented basal media to obtain the dose response curve for VEGF-dependent growth (Fig. 2F). The sunitinib IC_50_ for VEGF-dependent proliferation was 4.6 nM (95% CI, 2.6 nM, 8.0 nM), which is consistent with previous reports [20]. In comparison, the IC_50_ for CsA + Ita was roughly 700-fold higher, and was essentially unchanged from that observed in standard media. Thus, although less potent, the activity of CsA + Ita was independent of VEGF signaling.

### Cyclosporin A and Itraconazole Synergistically Inhibit Endothelial Tube Formation and Sprouting

Cell proliferation is a simplified measure of angiogenesis, representing only one aspect of angiogenesis *in vivo*. Alternative measures of drug efficacy against angiogenesis are the tube formation and sprouting assays, which entail cell migration, intercellular interactions, and differentiation. Thus, to complement the original screen, we utilized both these assays in further assessing the effect of the CsA + Ita combination. In the tube formation assay, HUVEC are grown on the surface of extracellular matrix on which they form a network of capillary-like structures. Under normal conditions, these networks are complex and highly branched, resembling capillary beds *in vivo*.

**Figure 3:**
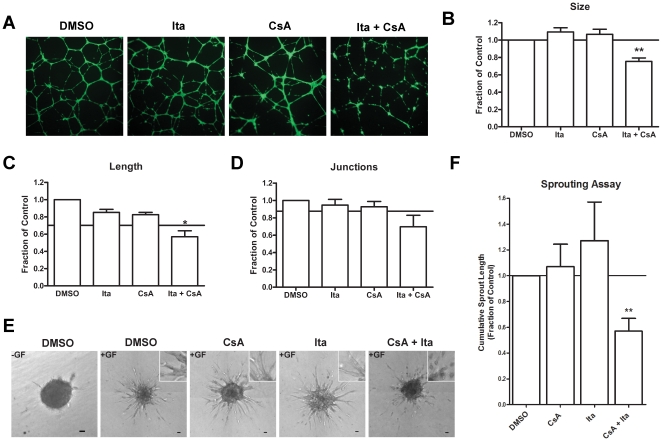
Cyclosporin A and itraconazole synergistically inhibit endothelial cell tube formation and sprout formation. (A) HUVEC were grown on a matrigel coated plates in the presence of vehicle only (DMSO), CsA (8 µM), Ita (800 nM), or a combination of the same CsA and Ita doses. The tube networks were stained with Calcein AM. Total tube length (B), number of junctions (C), and total network size (D) were calculated using AngioQuant (n = 3). (E) HUVEC spheres were embedded in a collagen matrix in the presence of basal media (EGM-2+2% FBS) (−GF), or complete media supplemented with VEGF_165_ and bFGF (+GF). The spheroids were treated with drug as in (A) (n = 4; scale bar = 50 µm). Insets are magnified views of the tube architecture. Cumulative sprout length was quantified (F). The non-interaction value is denoted by the horizontal line in B–D and F. Error bars, SEM; * p<0.1; ** p<0.05.

To test whether or not CsA and Ita synergized at the level of tube formation, HUVEC were seeded in a 24-well plate on a layer of pre-solidified matrigel in the presence or absence of CsA and Ita alone or in combination. After an 18-h treatment, the cells were stained to highlight the tube network and photographed (Fig. 3A). Using semi-automated software, several parameters of the tube network were quantified [15]. These included total tube size, which measures both the total length and thickness of the network, total tube length, which measures the linear length of the tubes, and lastly the number of junctions in the tube network (Fig. 3B–3D). The combination led to a decrease in tube network length, size, and junctions greater than that resulting from the single drugs alone.

The Chou-Talalay method for synergy analysis requires a complete dose-response curve for the single drugs. However, while it is relatively simple to generate a dose-response curve in the context of a proliferation assay, it is difficult to do so in a tube formation experiment since the distribution of any given drug between the matrix and the aqueous media is difficult to predict over the wide concentration range necessary. Thus, to analyze synergy in the tube formation assay, we utilized Webb's Fractional Product model, a modification of the Bliss Independence model for synergy, which does not require the full dose-response curve [24]–[26]. We found that for total network size the combination of CsA and Ita exhibited significant synergism (p<0.05). For total tube length, the synergism was more moderate in both degree and significance (p<0.1). In the case of junction formation, the combination trended towards synergy but did not reach significance. Together, these results suggest that the architecture of the network was not affected, but instead there was synergistic inhibition of the stability and viability of the tubes. For visual reference, the non-interaction value (i.e. the effect predicted by a simple additive interaction per Webb's Fractional Product model) is indicated by a horizontal line on Figure 3A–D. As a control, sunitinib and CsA + Ita were compared at several doses including the synergistic dose established in Figure 3 (Figure S2). Sunitinib significantly inhibited tube formation at 8.8 µM and 880 nM, whereas CsA + Ita only showed significant inhibition at the highest dose (8.8 µM). While sunitinib demonstrated greater potency in all parameters measures, these differences were mostly non-significant (Figure S2B).

In the sprout formation assay, spheres of endothelial cells were embedded in a three-dimensional collagen matrix and were induced to form sprouts by the application of growth factors including VEGF_165_ and bFGF. While neither CsA nor Ita alone were able to reduce cumulative sprout length, the CsA + Ita combination inhibited sprout formation by 43% (p<0.05) (Fig. 3E and 3F) and led the severe fragmentation of the sprouts. This was similar to the fragmentation observed in the tube formation assay. Thus, per Webb's Fractional Product model, CsA and Ita were also synergistic in the sprouting assay for angiogenesis.

### The combination of Cyclosporin A and Itraconazole does not cause general toxicity

**Figure 4:**
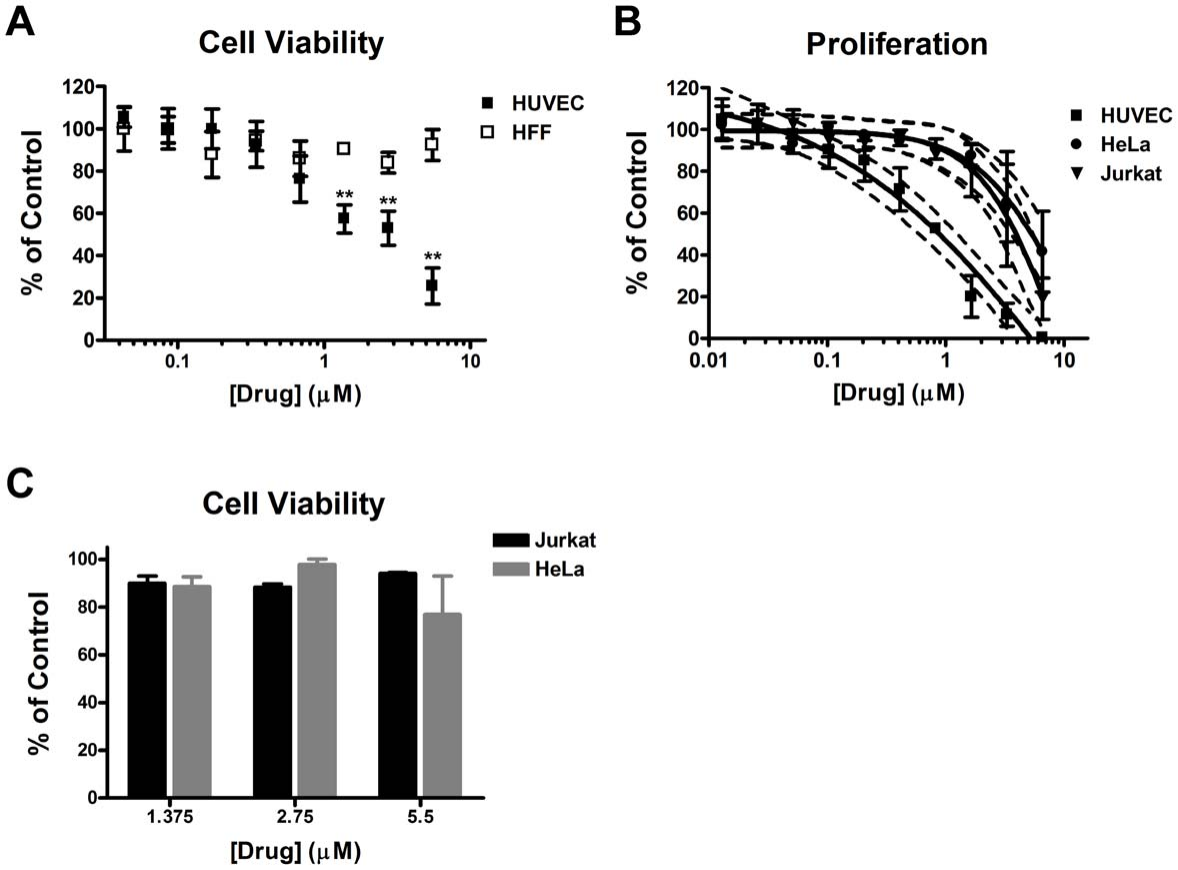
The combination of cyclosporin A and itraconazole does not cause general toxicity. (A) HUVEC and HFF were treated with a combination of CsA and Ita for 30 hours. Cell viability was measured by Calcein AM staining (n = 3). (B) Proliferation of HeLa and Jurkat T cells treated with a combination of CsA and Ita was compared to that of HUVEC after a 30 hour incubation (n = 3). Total combined drug dose shown (the ratio of CsA to Ita was 10∶1 as in other experiments). Bars, SEM; ** p<0.05; dashed lines indicate 95% confidence bands. (C) The viability of HeLa was determined as in (A) and Jurkat viability was determined after a 24 h drug dose followed by a 6 h incubation with Alamar blue. Bars = SEM; n = 3.

In some cases combining two drugs can lead to a synergistic toxicity as well as synergism of the desired effect. To evaluate whether or not general cellular toxicity was occurring in the case of the CsA-Ita combination, we compared the effects of the combination on the viability of HUVEC and another primary cell type, human foreskin fibroblasts (HFF) (Fig. 4A). We found that while the combination impaired the viability of HUVEC at higher doses, there was a negligible effect on the viability of HFF (Fig. 4A). In addition, we examined the effect of the combination on the proliferation of HeLa and Jurkat T cells in comparison to HUVEC (Fig. 4B). There was a window of roughly an order of magnitude between the potency of the combination against HUVEC and the other two cell types. The IC_50_ doses for HUVEC proliferation caused no effect on either HeLa or Jurkat T cells. In a subsequent experiment, the effect of CsA+Ita on HeLa and Jurkat viability was determined at the three highest doses used in the comparison of HUVEC and HFF viability. HeLa viability was reduced by less than 25% at the highest dose and by less than 12% at the lower two doses. Jurakat viability was not affect by more than 12% at any dose.

## Discussion

In this work, we took a novel approach to angiogenesis inhibition by screening a collection of clinically approved drugs for synergistic inhibition of endothelial cell proliferation. We undertook this approach both to circumvent the high cost involved in the development new drugs and to take advantage of the increase in potency resulting from synergism. Screening of 741 binary drug combinations resulted in the identification of a synergistic interaction between CsA and Ita in HUVEC proliferation, which was also observed in endothelial cell tube formation and sprouting assays. Importantly, this combination was selective for endothelial cells. To our knowledge, this is the first screen for synergy among clinical drugs for inhibition endothelial cell proliferation.

CsA is a natural product drug discovered in the 1970s and has been used widely as an immunosuppressant [27]. The ability of CsA to inhibit endothelial cell proliferation and angiogenesis has also been known for some time, but CsA has not been used as a clinical angiogenesis inhibitor due to its immunosuppressive properties and nephrotoxic side effects at high doses [28], [29]. Interestingly, while the mechanism for immunosuppression by CsA is due to inhibition of the protein phosphatase calcineurin in T cells, the antiangiogenic properties of the drug are independent of calcineurin [30], [31].

Itraconazole is a member of the triazole class of antifungal drugs. We previously identified itraconazole as an angiogenesis inhibitor in an initial screen of the JHDL [5]. Itraconazole inhibits lanosterol 14-α-demethylase (14DM), a key enzyme in the fungal ergosterol and human cholesterol biosynthetic pathways. However, it is unclear whether or not Ita can inhibit 14DM in humans and there is evidence that 14DM is not the relevant target for antiangiogenesis [32], [33]. We have shown that supplementation of HUVEC with cholesterol can only partially rescue the effects of itraconazole treatment and that there is a lack of correlation between fungal and endothelial cell proliferation for a series of stereoisomers of itraconazole [5], [34], [35]. Interestingly, itraconazole was shown by Heitman and colleagues to synergistically inhibit fungal growth with CsA, but this activity was shared by other azole antifungals suggesting that 14DM plays an important role in antifungal synergy [36]. In addition, we previously found that Ita inhibits mTOR in HUVEC and causes a defect in intracellular cholesterol trafficking [34].

Since its inception nearly four decades ago, the field of angiogenesis had been growing in complexity. While VEGF was the initial proangiogenic growth factor to be isolated, it is now known that multiple factors including FGF, PIGF, and PDGF all play roles in stimulating angiogenesis *in vivo*. Given that the induction of angiogenesis requires not only proliferation but also differentiation and migration, the complexity of signaling downstream of these growth factors is high. Although initially studied as independent units, there is now evidence that pro-angiogenic signaling pathways are integrative, branched networks of parallel pathways. This is evidenced by the finding that decreasing the expression levels of FGF and VEGF *in vivo* synergistically inhibited tumor angiogenesis [12]. Theoretically, such a network is a potential target for synergistic inhibition by a combination of small molecules which target different branches. The identification of CsA and Ita as synergistic inhibitors of endothelial cell proliferation, tube formation, and sprouting provides proof of principle for synergistic inhibition as a potential antiangiogenic strategy. The modest clinical effects of angiogenesis inhibitors like bevacizumab, which targets only a single node in the complex proangiogenic network, motivates pursuing a synergistic approach to antiangiogenesis [13]. That the potency of CsA+Ita against HUVEC proliferation was independent of VEGF signaling suggests an intriguing difference from bevacizumab, sunitnib, and other existing therapies.

In addition to the clinical implications, the identification of a synergistic drug combination may provide insight into the underlying molecular mechanisms of angiogenesis. Pharmacologic synergy is the chemical biology equivalent of synthetic lethality in classical genetics. Synthetic lethality implies that the two interacting alleles being tested work in either parallel arms of the same pathway or in two compensatory pathways [37]. Likewise, if the chemical perturbation of two distinct proteins by small molecules results in synergistic inhibition of angiogenesis, it may imply that the two protein targets are involved in similar or compensatory pathways responsible for angiogenesis. This knowledge can then be used to draw previously unexpected connections between pathways. A benefit of searching for synergy between clinical drugs is that a high proportion has known protein targets compared to non-drug chemical libraries. This increases the practicality of a mechanistic study of the observed synergy, which may potentially uncover novel interactions among angiogenesis signaling pathways.

In summary, we have provided proof of principle that identifying synergistic inhibition of endothelial cell proliferation between clinical drug combinations is possible in a high-throughput format. In addition the synergy between CsA and Ita has generated a launching off point to explore novel interactions between the relevant drug targets. Further screens of collections of drugs both with and without individual activity against HUVEC proliferation will likely lead to additional hits with potential clinical and basic science implications.

## Supporting Information

Figure S1 **Screening design.** In the first phase of screening, 741 combinations of 39 drugs were screened in duplicate using three doses centered around the IC_50_ of each drug. From this initial round, 47 combinations (6.3% preliminary hit rate) were identified as synergistic per a Chou-Talalay analysis. In the second phase, the initial hits were rescreened across a greater concentration range to generate full dose-response curves for the drugs both alone and in combination. The second phase produced one validated pair of clinical drugs, cyclosporin A and itraconazole (0.13% validated hit rate).
(EPS)

Figure S2 **Comparison of sunitinib and the cyclosporine A+itraconazole combination in tube formation assays.** (A) HUVEC were seeded on matrigel in the presence of the indicated compounds. Following an 18 h incubation, the tube networks were visualized with Calcein AM and photographed. Micrographs from one of four independent experiments are shown. (B) Total tube length, network size, and number of junctions were determined using Angioquant. Bars = SEM; n = 4; ** p<0.05; # p<0.005.
(TIF)
