# Supplementary figures and images for: Synergistic Inhibition of Endothelial Cell Proliferation, Tube Formation, and Sprouting by Cyclosporin A and Itraconazole
Source: PLoS One. 2011 Sep 28;6(9):e24793. doi: 10.1371/journal.pone.0024793 (PMC3182171; doi:10.1371/journal.pone.0024793)

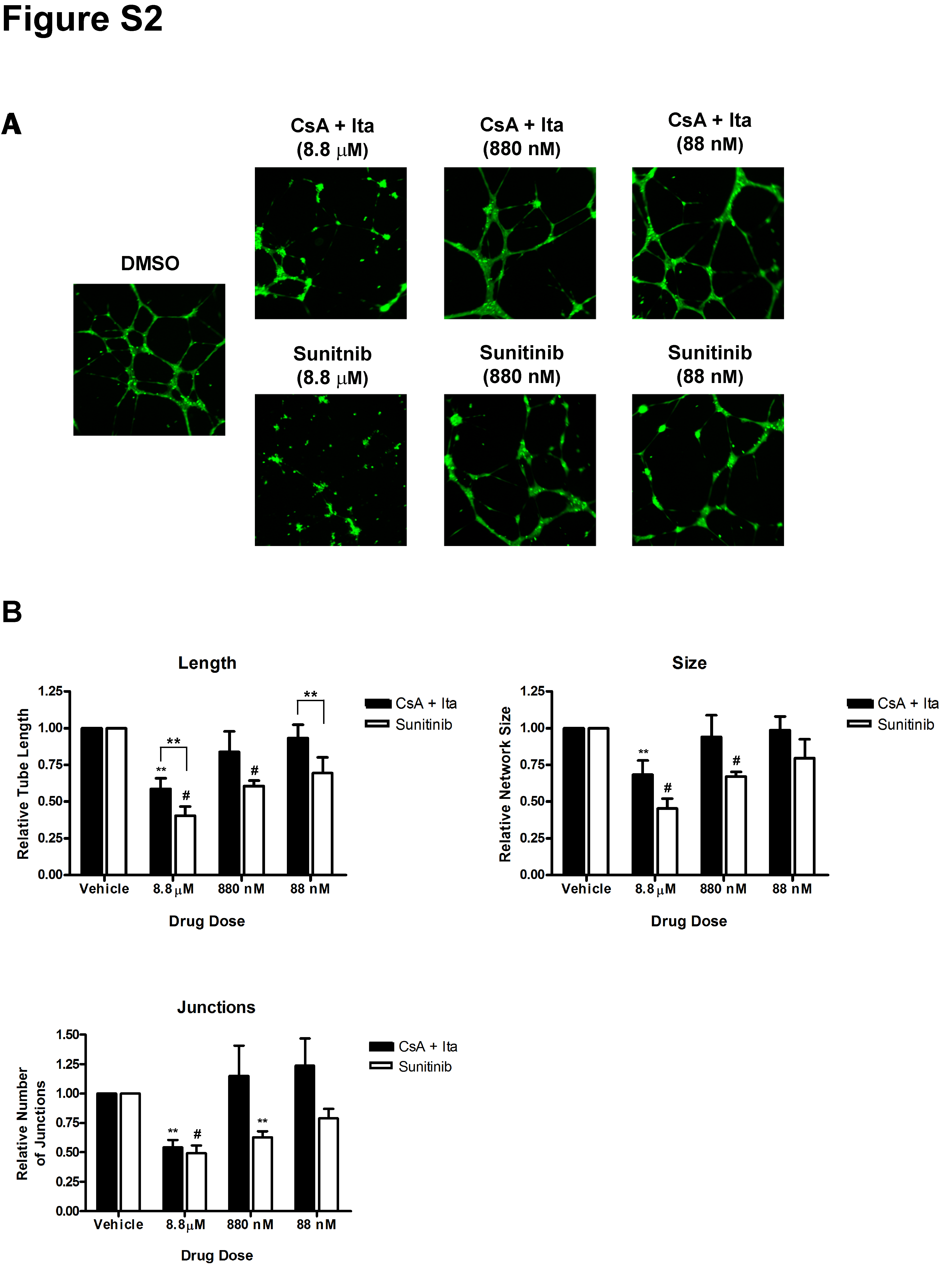

Supplement: Figure S2 — Comparison of sunitinib and the cyclosporine A+itraconazole combination in tube formation assays. (A) HUVEC were seeded on matrigel in the presence of the indicated compounds. Following an 18 h incubation, the tube networks were visualized with Calcein AM and photographed. Micrographs from one of four independent experiments are shown. (B) Total tube length, network size, and number of junctions were determined using Angioquant. Bars = SEM; n = 4; ** p<0.05; # p<0.005. (TIF) [file pone.0024793.s002.tif]
